# A quantitative, multi-national and multi-stakeholder assessment of barriers to the adoption of cell therapies

**DOI:** 10.1177/2041731417724413

**Published:** 2017-08-11

**Authors:** Benjamin M Davies, James Smith, Sarah Rikabi, Karolina Wartolowska, Mark Morrey, Anna French, Robert MacLaren, David Williams, Kim Bure, Rafael Pinedo-Villanueva, Anthony Mathur, Martin Birchall, Evan Snyder, Anthony Atala, Brock Reeve, David Brindley

**Affiliations:** 1 Division of Trauma and Orthopaedic Surgery, Department of Surgery, University of Cambridge, Cambridge, UK; 2 Nuffield Department of Orthopaedics, Rheumatology and Musculoskeletal Sciences, University of Oxford, Oxford, UK; 3 The UCL-Oxford Centre for the Advancement of Sustainable Medical Innovation, University of Oxford, Oxford, UK; 4 Department of Orthopedic Surgery, Mayo Clinic, Rochester, MN, USA; 5 Nuffield Laboratory of Ophthalmology, University of Oxford, Oxford, UK; 6 Centre for Biological Engineering, The Wolfson School of Mechanical, Electrical and Manufacturing Engineering, Loughborough University, Loughborough, UK; 7 Sartorius Stedim, Göttingen, Germany; 8 MRC Lifecourse Epidemiology Unit, University of Southampton, Southampton, UK; 9 NIHR Cardiovascular Biomedical Research Unit, London Chest Hospital, London, UK; 10 Department of Cardiology, Barts Health NHS Trust, London, UK; 11 Royal National Throat, Nose, and Ear Hospital, University College London, London, UK; 12 Sanford Burnham Prebys Medical Discovery Institute, San Diego, CA, USA; 13 Wake Forest Institute for Regenerative Medicine, Winston-Salem, NC, USA; 14 Harvard Stem Cell Institute, Cambridge, MA, USA; 15 Department of Paediatrics, University of Oxford, Oxford, UK; 16 Saïd Business School, University of Oxford, Oxford, UK; 17 Centre for Behavioural Medicine, UCL School of Pharmacy, University College London, London, UK

**Keywords:** Cell- and tissue-based therapy, regenerative medicine, stem cells, translational medical research, clinical adoption

## Abstract

Cellular therapies, such as stem cell–based treatments, have been widely researched and numerous products and treatments have been developed. Despite this, there has been relatively limited use of these technologies in the healthcare sector. This study sought to investigate the perceived barriers to this more widespread adoption. An anonymous online questionnaire was developed, based on the findings of a pilot study. This was distributed to an audience of clinicians, researchers and commercial experts in 13 countries. The results were analysed for all respondents, and also sub-grouped by geographical region, and by profession of respondents. The results of the study showed that the most significant barrier was manufacturing, with other factors such as efficacy, regulation and cost-effectiveness being identified by the different groups. This study further demonstrates the need for these important issues to be addressed during the development of cellular therapies to enable more widespread adoption of these treatments.

## Introduction

Cellular therapies and regenerative medicine are areas in which there has been rapid expansion in both research and the development of commercial products. These treatments seek to repair and regenerate damaged tissue in order to improve or restore function.^1^ Cellular therapies have been developed for use in a wide variety of medical specialities, a selection of which is outlined in Table 1. These products were asked about specifically in the questionnaire, having been selected at the time of pilot study as a representative sample of products that had marketing authorization and may be encountered by individuals in their professional capacities.

**Table 1.** Cellular therapy products asked about in questionnaire.

Product (producer)	Medical speciality	Target disease/disorder
ChondroCelect (TiGenix)	Orthopaedics	Knee cartilage defects
Cartistem (Medipost)	Orthopaedics	Osteoarthritis
Provenge (Dendreon)	Urology/Oncology	Metastatic prostate cancer
Carticel (Vericel)	Orthopaedics	Knee cartilage defects
Epicel (Vericel)	Plastic Surgery	Burns
Dermagraft (Organogenesis)	Podiatry	Diabetic foot ulcers
Apligraf (Organogenesis)	Vascular	Vascular and diabetic ulcers
Cupistem (Anterogen)	Colorectal Surgery	Anal fistulae
LaViv (Fibrocell Science)	Plastic Surgery	Nasolabial fold wrinkles
DCVax (Northwest Biotherapeutics)	Oncology	A variety of cancer types

Despite the development of these products and a large amount of spending on research and development, there has been relatively poor penetration of these products into the marketplace.^2^ For example, autologous chondrocyte implantation for the treatment of cartilage defects was first described over 20 years ago by Brittberg et al.,^3^ but it has not yet gained widespread adoption. This has been caused by a variety of factors including a lack of evidence demonstrating both the clinical and cost-effectiveness of the treatment,^4^ as well as the failure to identify the most appropriate target population in order to achieve best results.^5^

The majority of products listed have received marketing authorizations and/or accelerated patient access via US Breakthrough Status and/or European Medicines Agency Adaptive Pathways. In general, the marketing of these technologies is akin to other therapeutic platforms. However, for autologous therapeutics, one could postulate that a greater degree of clinical and/or patient involvement may be required compared to allogeneic approaches.

Our group sought to investigate the potential causes of this slow translation of research and early commercial development into widespread clinical adoption. We initially undertook a pilot study^6^ in which we asked a group of 50 clinicians to select the aspects that they felt were the greatest barriers to adoption from the following areas:

- Safety – a lack of proven safety data for these types of treatments;
- Efficacy (early) – a lack of Phase I/II trial data to justify conducting a larger trial;
- Efficacy (late) – a lack of cumulative clinical trial data to justify widespread adoption;
- Clinical trial methodologies – concerns regarding conduct and design of clinical trials to date;
- Cost-effectiveness – too costly for demonstrated benefit achieved;
- Usability – difficulties in use of products in clinical environment;
- Visibility/Lack of Knowledge – not previously aware of the availability of these treatments;
- Patient characteristics – products not applicable to majority of patient population;
- Patient attitudes and preferences – perceived dislike of cell therapies in patient population;
- Infrastructure – lack of clinical and laboratory facilities to make use of these products;
- Reimbursement – insufficient reimbursement to cover costs of providing treatment;
- Community – treatments not believed to be useful by other clinicians;
- Regulation – lack of approval for non-research use;
- Slow Progress – the slow pace of clinical trials;
- Research Cost – inhibition of investigator-led research due to high costs;
- Manufacturing issues – difficulties in manufacture, scale-up problems and cost.

The most important factors identified by the pilot study as preventing the more widespread adoption of cellular therapies were cost-effectiveness, efficacy and reimbursement issues. The primary limitation of our pilot study, affecting the generalizability of our findings, was its restriction to clinicians in the United Kingdom.

Recently, other researchers have sought to help improve the process for cellular therapy development. Sridharan et al.^7^ have thoroughly outlined a potential pathway that can be used in the development of new treatments for cartilage defects. They highlighted the importance of having a well-defined pathway for the development of translational products in order to improve the flow of products into clinical practice. Despite this, the issues that we identified in our pilot study still seem to be a cause for the failure of successful product development.^8^

We therefore sought to repeat the study using a wider spread of professional groups and without any geographical limitations.

## Methods

The questionnaire used was developed from the previous pilot study. The study received ethical approval from our institutional review board and used an implied consent model. The final questionnaire was formatted into an online form for easy distribution and completion. A full copy of the questionnaire can be found in Supplementary Material S1.

Participants were identified from mailing lists held by CASMI and attendees at appropriate conferences. They were invited to complete an anonymous questionnaire via email in the first half of 2016.

Participants were asked a number of questions about their experience of cellular therapies, their professional background (clinical, researcher, commercial) and how important different barriers were to the clinical adoption of cellular therapies. Some questions varied by job response to allow tailoring of questions to an individual’s experience. For example, when asked about experience of using cellular therapeutics, clinicians were asked if they had used a cellular therapy clinically whereas scientists were asked if they were involved in research into cellular therapeutics. When asked to assess different potential barriers to the adoption of cellular therapies, the different barriers were presented in a random order which changed between participants to avoid any bias due to question order.

Results were analysed for the whole group and for the different professional subgroups. Statistical analysis was undertaken in GraphPad Prism 6.0h (La Jolla, CA, USA).

Although the extent to which each of the 16 aspects of interest were perceived to be barriers to the adoption of cellular therapies was assessed with questions offering five categories as possible answers, we converted these to discrete numerical values in order to summarize average response levels. These ranged from 1 if the answer was ‘No barrier’ to 5 if it was ‘Significant barrier’. These numerical values were then used to create weighted averages to indicate which barriers were thought to be most significant.

We used the Tukey–Kramer test to determine which groups (Clinicians, Researchers, Commercial) differed significantly from one another in assessment of barriers. As normality is assumed for this test, we confirmed it using the D’Agostino and Pearson omnibus normality test. This test allows for different sized groups, including those with low numbers.^9^

## Results

**Figure 1.**
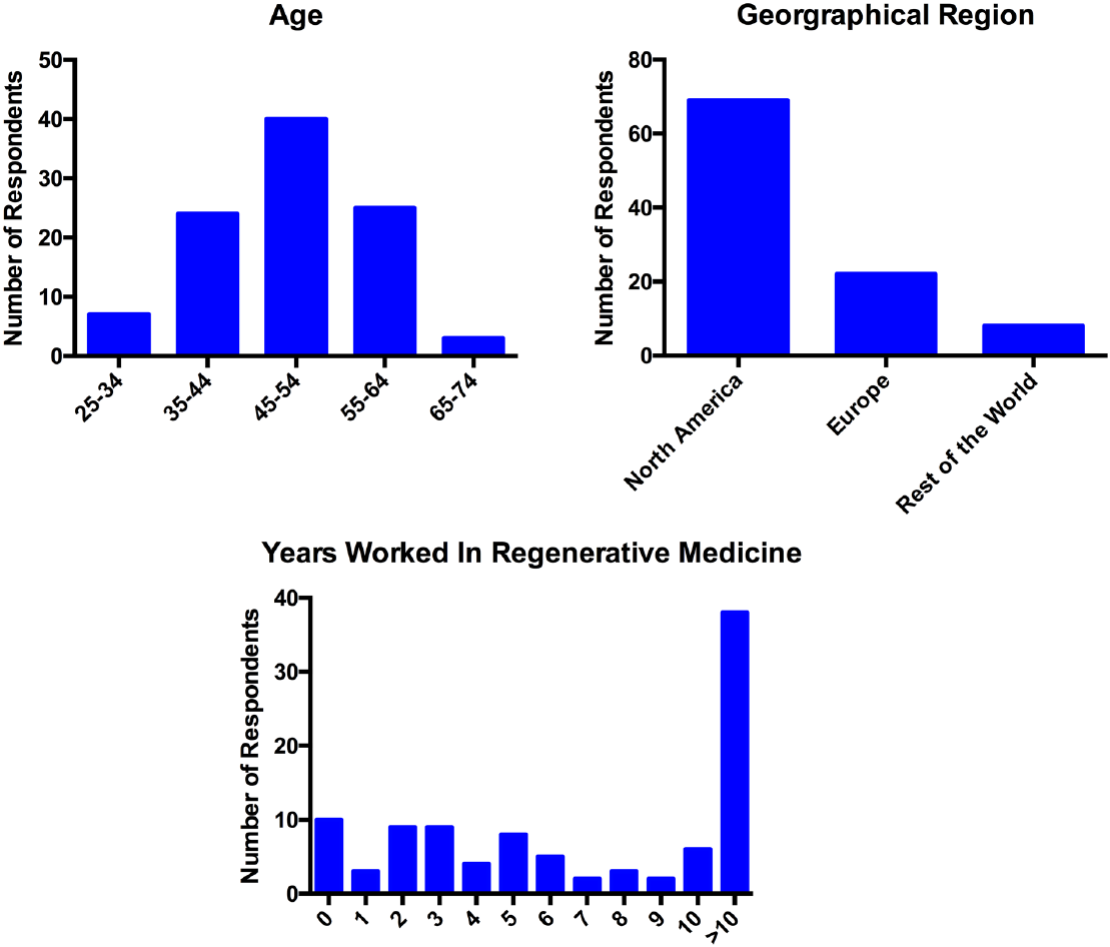
Demographic information for all respondents (n = 99).

The survey was completed by 131 people. However, a number of individuals submitted incomplete surveys; therefore, 99 complete responses were available for analysis. The median age band range of respondents was 45–54 years. The graphs in Figure 1 show the demographic information for the respondents.

For the purpose of further analysis, the respondents were split into three groups based on their reported job role as outlined below. Individuals who responded ‘Other’ for their job role had their text response analysed to allow allocation to one of the groups listed below (data not shown):

- ‘Clinician’ included Scientist – Clinician and Medical Doctors (n = 8);
- ‘Researcher’ included Scientist – Academia and Scientist – Industry (n = 36);
- ‘Commercial’ included Commercial – Strategic Partnership, Commercial – Other, and Regulatory Professional (n = 55).

Clinicians came from a wide variety of different specialities including cardiology, ophthalmology, oncology, haematology and transplantation medicine.

**Figure 2.**
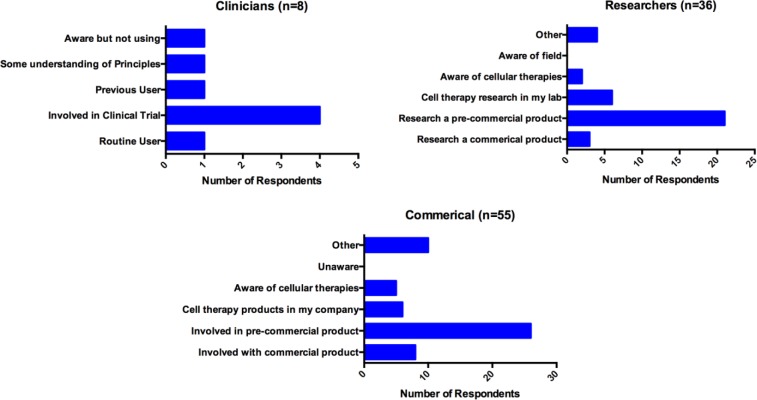
Experience of cellular therapies by job type.

The majority of respondents had some knowledge of the use of cellular therapeutics. Figure 2 shows the responses provided by the different groups. A small number of individuals did not answer this question, hence the change in numbers in each group.

When asked about their experience of a range of commercially available cellular therapy products, the majority of respondents had an awareness of, but no direct experience of the products mentioned. This fits with the majority of individuals’ experience being in the development of as yet unreleased products.

**Figure 3.**
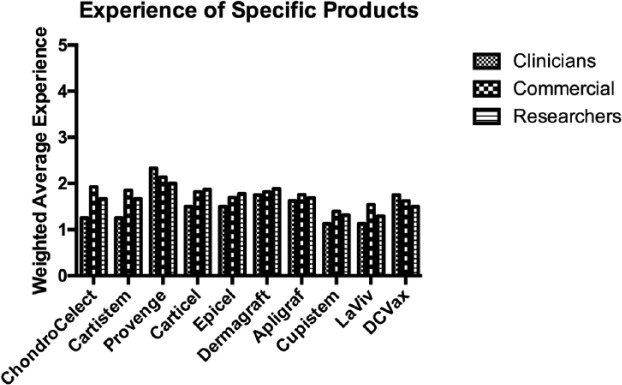
Experience of specific cellular therapy products. (Results are presented as a weighted average where 5 would equate to direct experience of the product and 1 would indicate no awareness of the product.)

Figure 3 shows the experience of individuals of each product (a weighted average score was produced for each product by converting the responses to numerical values).

**Figure 4.**
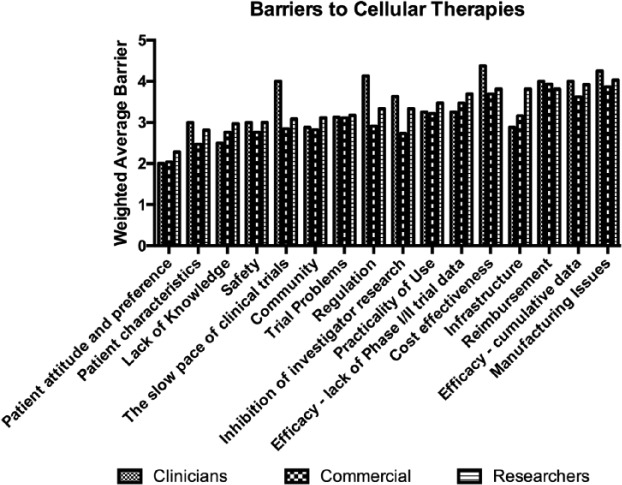
Perceived barriers to the adoption of cellular therapies. (Results are presented as a weighted average where 5 would equate to a significant barrier and 1 would indicate no barrier.)

Figure 4 shows the mean converted numerical level of barrier for all aspects by group. More detailed results showing the number of each type of response are provided in Supplementary Material 2.

As can be seen, there was a generally reasonable level of agreement as to what barriers were significant between the commercial group and researchers. For the whole group, the most significant barrier was felt to be manufacturing, followed by reimbursement, and cost-effectiveness. Table 2 shows the three greatest perceived barriers as identified by each group according to the converted numerical mean score.

**Table 2.** The three biggest barriers to adoption as identified by each group.

Barrier ranking	Clinicians (n = 8)	Commercial (n = 55)	Researchers (n = 36)
1	Cost-effectiveness	Reimbursement	Manufacturing
2	Manufacturing	Manufacturing	Efficacy (cumulative data)
3	Regulation	Cost-effectiveness	Reimbursement

We also looked at the effect of region on responses. Table 3 shows the biggest barriers to adoption as perceived in different geographical regions.

**Table 3.** The three biggest barriers to adoption as identified by different geographical regions.

Barrier ranking	North America (n = 69)	Europe (inc. UK) (n = 22)	Rest of the World (n = 8)
1	Manufacturing	Cost-effectiveness	Reimbursement
2	Reimbursement	Reimbursement	Manufacturing
3	Efficacy (cumulative data)	Manufacturing	Efficacy (Phase I/II trials)

The barriers which were significant at the 0.05 level were ‘The slow pace of clinical trials’ and ‘Regulation’ between Clinicians and Commercial, and ‘Inhibition of investigator research’ and ‘Infrastructure’ between Researchers and Commercial.

## Discussion

These results show that the primary issues that are believed to prevent the more widespread adoption of cellular therapies remain relatively constant when a wider spread of professional groups is surveyed. The identification of manufacturing issues as of primary importance may well be related to the inclusion of individuals from the commercial sector who have greater exposure to, and understanding of, the issues related to product development and production as compared to clinicians. The importance of manufacturing issues appears to be becoming more clearly appreciated with it being recognized in this study to a much greater extent than in the pilot study.^6^

Compared to the pilot study,^6^ manufacturing displaced efficacy as the second greatest barrier from the viewpoint of clinicians. The complex nature of biomanufacturing and distribution processes, and the associated costs, have been highlighted by others as a key reason for the need of these treatments to demonstrate their superior efficacy over current treatments in order to successfully enter the marketplace.^10^

The highlighting of these issues demonstrates that although there is a large amount of development on cellular therapies taking place in both the academic and commercial fields as shown by the number of registered clinical trials utilizing stem cells,^11^ there has not been sufficient work done to address all of the concerns which may prevent widespread adoption. If products are to achieve widespread use in the clinical environment, the issues highlighted by this study must be addressed. The difficulties of manufacturing cellular therapies are significant and overcoming them may limit adoption of these products. However, as a wider and wider variety of cellular therapies are developed, it should be possible for the adoption of common pathways and processes to help reduce cost and complexity in product design and development.

Product development cycles, from initial concept to widespread clinical roll-out, should be designed in order to ensure that the concerns of all groups are addressed.^7^ The importance of aligning the regulatory processes and reimbursement strategies to suit all stakeholders is vital to this process and has been identified by national bodies such as the National Institute for Health and Care Excellence (NICE, UK) and the Centre for Commercialization of Regenerative Medicine (CCRM, Canada).^12^ This will, hopefully, enable the increased adoption of these products into the clinical setting.

These issues are becoming more and more prominent as demonstrated by a recent opinion piece by Caplan et al.^13^ This article highlights the importance of ‘regulation, reimbursement, and realization of value’. The first two elements are represented highly in our responses, while the third demonstrates the issues surrounding the uncertainty of how much these products are ‘worth’, given their high development cost and unpredictable market adoption and clinical success. This further emphasizes the importance of the factors that have been highlighted in this article.

The study is limited by having been designed as a voluntary survey with no follow-up of non-respondents. This may have led to some bias in the responses received. The respondents were also drawn mainly from North America, which may have led to responses more affected by the healthcare model in North America than that found in the United Kingdom and other parts of Europe. It should also be highlighted that the majority of both commercial and research respondents are involved in the development of products that have not yet made it to market. While this may reflect the nature of the cellular therapies marketplace, it may have an impact on their views and hence responses. Lack of experience of products further down the development path may have led them to particularly underestimate some of the barriers that can be encountered during widespread deployment of a product, such as product storage and use, regulatory approvals and patient acceptability.

The lack of experience of respondents to particular cellular therapies may, initially, seem surprising. However, we believe that there are two possible explanations for this. First, the cellular therapy industry is, in comparison to the majority of other areas of healthcare, in its nascency and of relatively small size. This will have limited the possible exposure of individuals to the field. Second, a high proportion of currently available cellular therapies are designed for orphan, or non-mainstream, clinical indications. This further limits the likelihood of individuals coming across particular products unless directly involved in their use.

A further issue, which was not specifically asked about in the questionnaire, but which may prove a further barrier, is the complexity of providing a suitable clinical trial site, with requisite capacity, skills and resources to support cell-based therapy trials. Lack of experience among respondents to this aspect of product development may also explain why it was not raised by any of them. The logistics involved in product collection, storage and delivery, which may require maintenance of sterility can be complex in a healthcare environment. For example, where initial sample collection may occur in an operating theatre and/or leukapheresis facility. Additionally, some elements of product final formulation and finish may occur in hospital pharmacies.

Obtaining responses from a cross section of those involved in the development of cellular therapy products has enabled us to produce an overview of the issues that are felt to affect the whole development cycle of these treatments. It should be noted that only eight clinicians were included in this study. This does provide a significant limitation in the extrapolation of results. However, our pilot study contained 50 responses gathered purely from clinicians with some indicators of inter-respondent response consistency noted.^6^ The results of this study should therefore be looked at with reference to the pilot study to obtain the full picture of opinion across the sector.

## Conclusion

This study shows that in order to drive forward the adoption of cellular therapies the development of future products should aim to address the entire life cycle of development from the earliest of stages to their clinical implementation. Addressing down-stream concerns such as manufacturing scale-up and the practicality of product supply is vital to its success. We believe that the development of standards in the process of cellular therapy product development will help in this regard.

## Supplementary Material

Supplementary material

Supplementary material
