# Supplementary material for: A quantitative, multi-national and multi-stakeholder assessment of barriers to the adoption of cell therapies
Source: J Tissue Eng. 2017 Aug 11;8:2041731417724413. doi: 10.1177/2041731417724413 (PMC5557158; doi:10.1177/2041731417724413)
Supplement: Supplementary material [file Supplimentary_Material_S1.pdf]

CASMI

Introduction

Thank you for agreeing to take part in this survey. CASMI (The Centre for the Advancement of Sustainable Medical Innovation, a collaboration between University College London and the University of Oxford) is investigating the issues that are preventing the transition of cellular therapeutics into the clinical environment.

We would be very grateful if you could answer the following questions to help us better understand the most important problems in this area. You will be asked about your previous exposure to cellular therapies and then asked to rank a number of potential barriers. The survey should take no more than five to ten minutes to complete. Taking part is entirely voluntary and all responses are collected anonymously. The results will be collated and published in a peer reviewed journal in due course. Survey results will be collated within SurveyMonkey anonymously and the results held by the University of Oxford for 5 years.

If you are still happy to take part, please click on the "Next" button below.

Many thanks,

Benjamin Davies and David Brindley, on behalf of CASMI.

This project has been reviewed by, and received ethics clearance through, the University of Oxford Central University Research Ethics Committee. If you have a concern about any aspect of this project, please contact Dr Brindley at david.brindley@sbs.ox.ac.uk and he will do his best to answer your query. The researcher should acknowledge your concern within 10 working days and give you an indication of how he intends to deal with it. If you remain unhappy or wish to make a formal complaint, please contact the chair of the Research Ethics Committee at the University of Oxford (using the contact details below) who will seek to resolve the matter in a reasonably expeditious manner:

Chair, Social Sciences & Humanities Inter-Divisional Research Ethics Committee; Email: ethics@socsci.ox.ac.uk; Address: Research Services, University of Oxford, Wellington Square, Oxford OX1 2JD)

Partners:

CASMI

Demographic Information

* What is your age?

- ◯ 18 to 24
- ◯ 25 to 34
- ◯ 35 to 44
- ◯ 45 to 54
- ◯ 55 to 64
- ◯ 65 to 74
- ◯ 75 or older

* Please list the country you work in.

[ ]

CASMI

Sector

* What is your job?

- ◯ Medical Doctor
- ◯ Scientist - Academia
- ◯ Scientist - Industry
- ◯ Commercial - Strategic Partnership
- ◯ Commercial - Other
- ◯ Scientist - Clinician
- ◯ Regulatory Professional
- ◯ Other

* How many years have you worked in this field?

* How many years have you worked specifically in Regenerative Medicine?

CASMI

Other Profession

Please enter your profession in the box below.

Clinical Speciality

* What speciality do you work in?

- ◯ Cardiology
- ◯ Neurology
- ◯ Ophthalmology
- ◯ Orthopedic Surgery
- ◯ Plastic and Reconstructive Surgery
- ◯ Urology
- ◯ Other (please specify)

CASMI

Clinical Exposure to Cellular Therapeutics and Mesenchymal Stem Cells

* Please choose the answer that best describes your experience of using cellular therapeutics.

- ◯ Routine user of cellular therapies
- ◯ Currently holding or planning a cell therapy clinical trial
- ◯ A previous user of cellular therapies; however, for any reason(s) have ceased using them (please state why on next page).
- ◯ Some understanding the fundamental science underpinning cellular therapeutics
- ◯ Aware of available cellular therapies in your field; however, for specific reason(s) unwilling to use them (please state why on next page).
- ◯ Unaware of landscape and possible cellular therapy options
- ◯ Other (please specify)

CASMI

Specific Reason(s)

Specific reason(s) for not using cellular therapy products

CASMI

Specific Reason(s) for stopping

Specific reason(s) for stopping using cellular therapy products

CASMI

Research Exposure to Cellular Therapies

* Please choose the answer that best describes your experience of using cellular therapeutics.

- ◯ Involved in research for commercially available product
- ◯ Involved in research for product not yet commercially available
- ◯ Research into cellular therapies taking place in my lab
- ◯ Aware of cellular therapies but not involved in any product development
- ◯ Aware of field from journals/media etc
- ◯ Other (please specify)

CASMI

Industry and Commerical Exposure to Cellular Therapies

* Please choose the answer that best describes your experience of using cellular therapeutics.

- ◯ Involved in development and/or marketing of commercially available product
- ◯ Involved in development and/or marketing of product not yet commercially available
- ◯ Work for company with cellular therapy product but not directly involved in their sale or development
- ◯ Aware of cellular therapy products but no direct involvement
- ◯ Unaware of the field
- ◯ Other (please specify)

CASMI

Other Exposure to Cellular Therapies

* Please choose the answer that best describes your experience of using cellular therapeutics.

- ◯ Professional involved in the development or design of a commercially available product
- ◯ Professional involved in the development or design of a product not yet commercially available
- ◯ Work for company with a cellular therapy product but not directly involved in their sale or development
- ◯ Aware of cellular therapy products but no direct involvement
- ◯ Unaware of the field
- ◯ Other (please specify)

CASMI

Experience of Cell Therapy Products - Clinical

For each of the products listed below please select the answer that most closely matches your experience in using the product.

* Select the most appropriate answer for each product.

	Use Regularly	Used in the past	Encountered in a clinical setting	Encountered in media/journals	Not aware of this product
ChondroCelect (TiGenix) - for the treatment of knee cartilage defects.	○	○	○	○	○
Cartistem (Medipost) - for the treatment of traumatic and degenerative osteoarthritis.	○	○	○	○	○
Provenge (Dendreon) (sipuleucel-T) - for the treatment of metastatic castrate-resistant prostate cancer.	○	○	○	○	○
Carticel (Vericel - previously Genzyme Biosurgery/Sanofi-Genzyme/Aastrom) - for the treatment of knee cartilage defects.	○	○	○	○	○
Epicel (Vericel - previously Genzyme Biosurgery/Sanofi-Genzyme/Aastrom) - for the treatment of deep dermal or full thickness burns.	○	○	○	○	○
Dermagraft (Organogenesis) - for the treatment of diabetic foot ulcers.	○	○	○	○	○
Apligraf (Organogenesis) - for the treatment of chronic venous leg ulcers and diabetic foot ulcers.	○	○	○	○	○
Cupistem (Anterogen) - for the treatment of anal fistulas.	○	○	○	○	○
LaViv (Fibrocell Science) (azficel-T) - for the treatment of nasolabial fold wrinkles	○	○	○	○	○
DCVax (Northwest Biotherapeutics) - for the treatment of a variety of cancers	○	○	○	○	○

CASMI

Experience of Cell Therapy Products - Research

For each of the products listed below please select the answer that most closely matches your experience with this product.

* Select the most appropriate answer for each product.

	Directly Studied	Clinical trial undertaken in my organisation	Laboratory studies undertaken in my organisation	Encountered in media/journals	Not aware of this product
ChondroCelect (TiGenix) - for the treatment of knee cartilage defects.					
Cartistem (Medipost) - for the treatment of traumatic and degenerative osteoarthritis.					
Provenge (Dendreon) (sipuleucel-T) - for the treatment of metastatic castrate-resistant prostate cancer.					
Carticel (Vericel - previously Genzyme Biosurgery/Sanofi-Genzyme/Aastrom) - for the treatment of knee cartilage defects.					
Epicel (Vericel - previously Genzyme Biosurgery/Sanofi-Genzyme/Aastrom) - for the treatment of deep dermal or full thickness burns.					
Dermagraft (Organogenesis) - for the treatment of diabetic foot ulcers.					
Apligraf (Organogenesis) - for the treatment of chronic venous leg ulcers and diabetic foot ulcers.					
Cupistem (Anterogen) - for the treatment of anal fistulas.					
LaViv (Fibrocell Science) (azficel-T) - for the treatment of nasolabial fold wrinkles					
DCVax (Northwest Biotherapeutics) - for the treatment of a variety of cancers					

CASMI

Experience of Cell Therapy Products - Industry

For each of the products listed below please select the answer that most closely matches your experience with this product.

* Select the most appropriate answer for each product.

	Involved in development/marketing	Developed/marketed in my organisation	Encountered in media/journals	Not aware of this product
ChondroCelect (TiGenix) - for the treatment of knee cartilage defects.	○	○	○	○
Cartistem (Medipost) - for the treatment of traumatic and degenerative osteoarthritis.	○	○	○	○
Provenge (Dendreon) (sipuleucel-T) - for the treatment of metastatic castrate-resistant prostate cancer.	○	○	○	○
Carticel (Vericel - previously Genzyme Biosurgery/Sanofi-Genzyme/Aastrom) - for the treatment of knee cartilage defects.	○	○	○	○
Epicel (Vericel - previously Genzyme Biosurgery/Sanofi-Genzyme/Aastrom) - for the treatment of deep dermal or full thickness burns.	○	○	○	○
Dermagraft (Organogenesis) - for the treatment of diabetic foot ulcers.	○	○	○	○
Apligraf (Organogenesis) - for the treatment of chronic venous leg ulcers and diabetic foot ulcers.	○	○	○	○
Cupistem (Anterogen) - for the treatment of anal fistulas.	○	○	○	○
LaViv (Fibrocell Science) (azficel-T) - for the treatment of nasolabial fold wrinkles	○	○	○	○
DCVax (Northwest Biotherapeutics) - for the treatment of a variety of cancers	○	○	○	○

CASMI

Experience of Cell Therapy Products - Other

For each of the products listed below please select the answer that most closely matches your experience with this product.

* Select the most appropriate answer for each product.

	Involved in design or development	Developed/designed in my organisation	Encountered in media/journals	Not aware of this product
ChondroCelect (TiGenix) - for the treatment of knee cartilage defects.	○	○	○	○
Cartistem (Medipost) - for the treatment of traumatic and degenerative osteoarthritis.	○	○	○	○
Provenge (Dendreon) (sipuleucel-T) - for the treatment of metastatic castrate-resistant prostate cancer.	○	○	○	○
Carticel (Vericel - previously Genzyme Biosurgery/Sanofi-Genzyme/Aastrom) - for the treatment of knee cartilage defects.	○	○	○	○
Epicel (Vericel - previously Genzyme Biosurgery/Sanofi-Genzyme/Aastrom) - for the treatment of deep dermal or full thickness burns.	○	○	○	○
Dermagraft (Organogenesis) - for the treatment of diabetic foot ulcers.	○	○	○	○
Apligraf (Organogenesis) - for the treatment of chronic venous leg ulcers and diabetic foot ulcers.	○	○	○	○
Cupistem (Anterogen) - for the treatment of anal fistulas.	○	○	○	○
LaViv (Fibrocell Science) (azficel-T) - for the treatment of nasolabial fold wrinkles	○	○	○	○
DCVax (Northwest Biotherapeutics) - for the treatment of a variety of cancers	○	○	○	○

CASMI

Clinical Barriers to the Adoption of Cellular Therapeutics

In the question below we would be grateful if you could rank each factor as to how signficant a barrier you feel it to be to the adoption of cellular therapies. These areas have previously been identified as potential problems for product adoption in a previous pilot study which is available via Open Access here (Davies, B. M. et al. Quantitative assessment of barriers to the clinical development and adoption of cellular therapies: A pilot study. J Tissue Eng 5, 2041731414551764 (2014))

* Evaluate the following statements.

	Not a barrier to adoption		A moderate barrier to adoption		A signficant barrier to adoption
Safety - lack of proven safety data for these types of treatments.	○	○	○	○	○

Please list any specfic reasons for your response.

	Not a barrier to adoption		A moderate barrier to adoption		A signficant barrier to adoption
Efficacy - lack of Phase I/II trial data to justify conducting a larger trial.	○	○	○	○	○

Please list any specfic reasons for your response.

	Not a barrier to adoption		A moderate barrier to adoption		A signficant barrier to adoption
Efficacy - lack of cumulative clinical trial data to justify widespread adoption.	○	○	○	○	○

Please list any specfic reasons for your response.

	Not a barrier to adoption		A moderate barrier to adoption		A signficant barrier to adoption
Trial Problems - concerns regarding conduct and design of clinical trials to date.	○	○	○	○	○

Please list any specfic reasons for your response.

	Not a barrier to adoption		A moderate barrier to adoption		A signficant barrier to adoption
Cost effectiveness - too costly for demonstrated benefit achieved.	○	○	○	○	○

	Not a barrier to adoption		A moderate barrier to adoption		A signficant barrier to adoption

Please list any specfic reasons for your response.

Practicality of Use - difficulties in use of products in clinical environment.					

Please list any specfic reasons for your response.

Lack of Knowledge - not previously aware of the availability of these treatments.					

Please list any specfic reasons for your response.

Patient characteristics - products not applicable to majority of patient population.					

Please list any specfic reasons for your response.

Patient attitude and preference - perceived dislike of cell therapies in patient population.					

Please list any specfic reasons for your response.

Infrastructure - lack of clinical and laboratory facilities to make use of these products.					

Please list any specfic reasons for your response.

Reimbursement - insufficient reimbursement to cover costs of providing treatment.					

	Not a barrier to adoption		A moderate barrier to adoption		A signficant barrier to adoption

Please list any specfic reasons for your response.

Community - treatments not believed to be useful by other clinicians.

Please list any specfic reasons for your response.

Regulation - lack of approval for non-research use.

Please list any specfic reasons for your response.

The slow pace of clinical trials

Please list any specfic reasons for your response.

Inhibition of investigator led research due to high costs

Please list any specfic reasons for your response.

Manufacturing Issues - difficulties in manufacture, scale-up problems, and cost.

Please list any specfic reasons for your response.

Please detail any other barriers that you feel are important in the box below.

CASMI

Thank you for taking part in this survey. Your responses will prove extremely valuable in our attempts to further understand the barriers to clinical adoption of cellular therapeutics.

If you have any further questions, please feel free to contact us.

More information can be found on the CASMI Translational Stem Cell Consortium Webpage
