# Supplementary material for: A quantitative, multi-national and multi-stakeholder assessment of barriers to the adoption of cell therapies
Source: J Tissue Eng. 2017 Aug 11;8:2041731417724413. doi: 10.1177/2041731417724413 (PMC5557158; doi:10.1177/2041731417724413)
Supplement: Supplementary material [file Supplimentary_Material_S2.pdf]

Supplementary Material S2 – Breakdown of results by each type of response to questions on barriers to cellular therapies

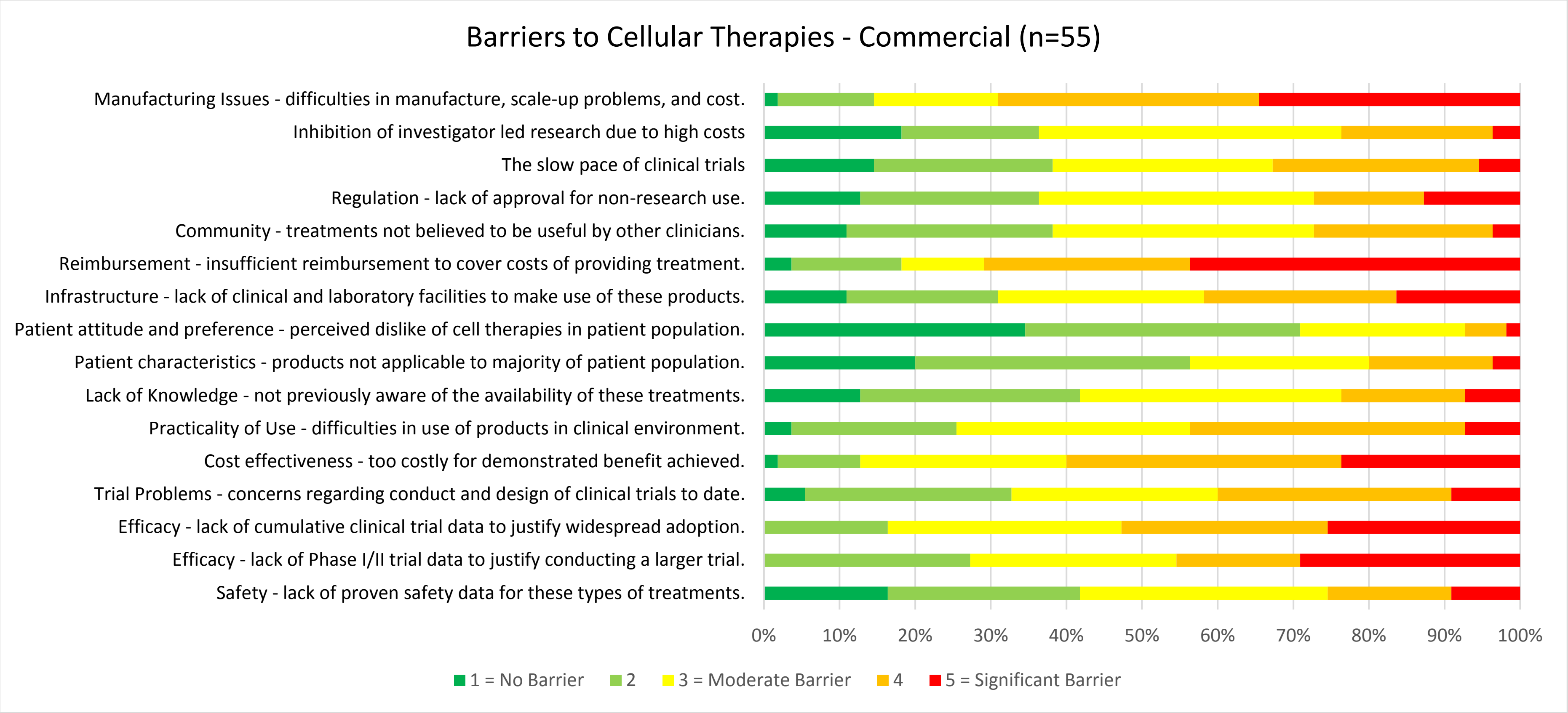

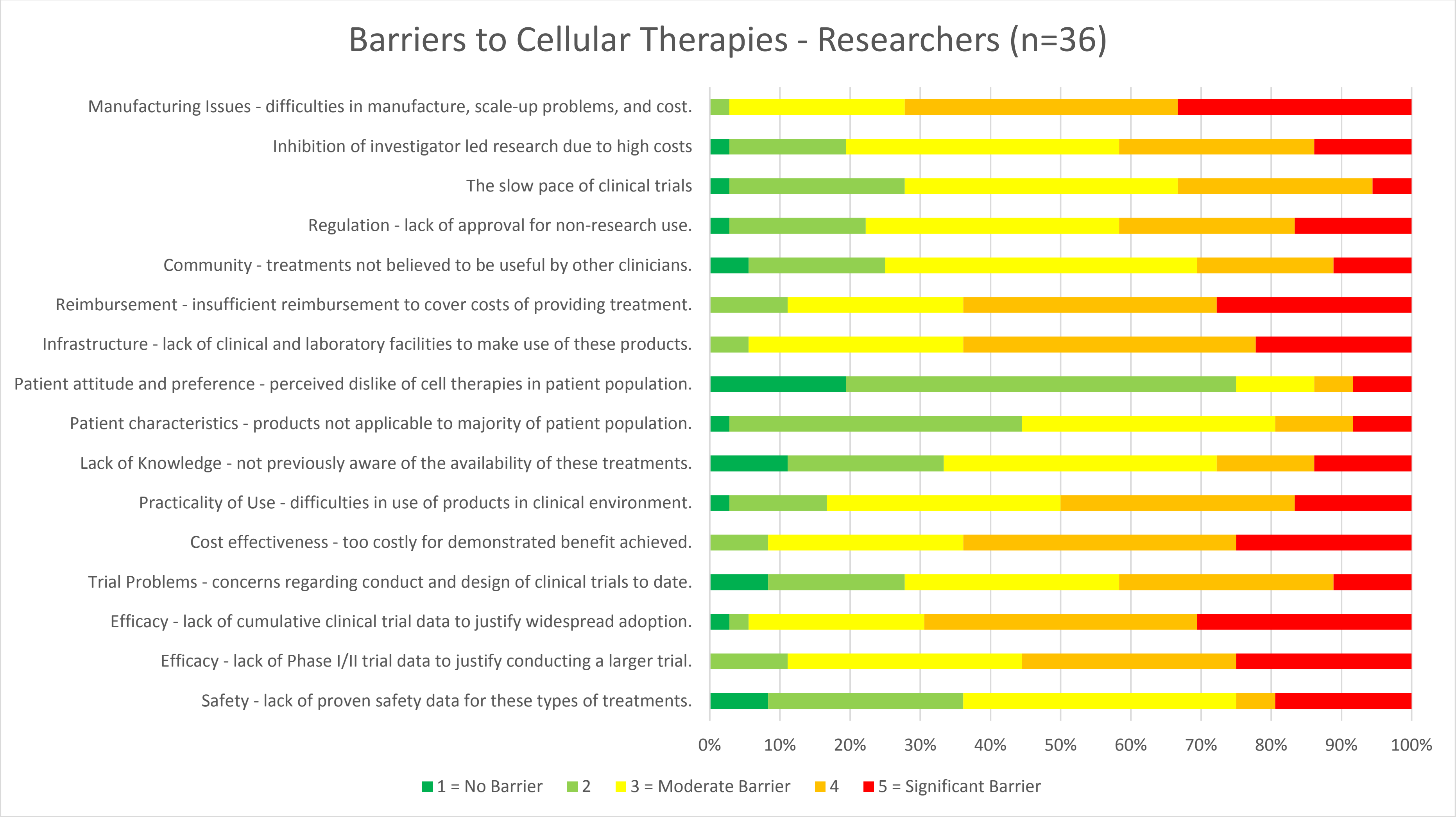
Barriers to Cellular Therapies - Researchers (n=36)
Manufacturing Issues - difficulties in manufacture, scale-up problems, and cost.
Inhibition of investigator led research due to high costs
The slow pace of clinical trials
Regulation - lack of approval for non-research use.
Community - treatments not believed to be useful by other clinicians.
Reimbursement - insufficient reimbursement to cover costs of providing treatment.
Infrastructure - lack of clinical and laboratory facilities to make use of these products.
Patient attitude and preference - perceived dislike of cell therapies in patient population.
Patient characteristics - products not applicable to majority of patient population.
Lack of Knowledge - not previously aware of the availability of these treatments.
Practicality of Use - difficulties in use of products in clinical environment.
Cost effectiveness - too costly for demonstrated benefit achieved.
Trial Problems - concerns regarding conduct and design of clinical trials to date.
Efficacy - lack of cumulative clinical trial data to justify widespread adoption.
Efficacy - lack of Phase I/II trial data to justify conducting a larger trial.
Safety - lack of proven safety data for these types of treatments.
0%
10%
20%
30%
40%
50%
60%
70%
80%
90%
100%
1 = No Barrier
2
3 = Moderate Barrier
4
5 = Significant Barrier

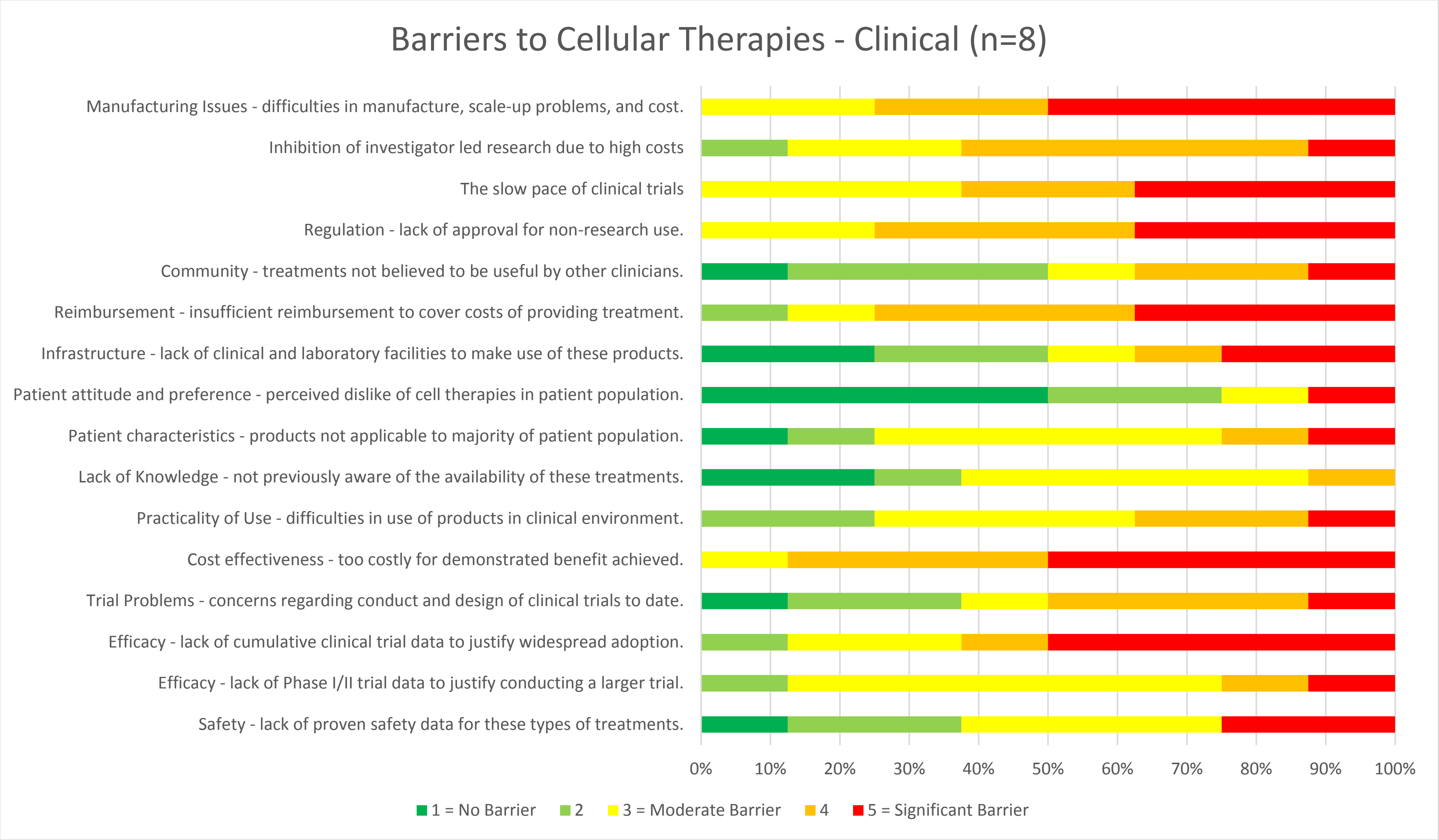
Barriers to Cellular Therapies - Clinical (n=8)
Manufacturing Issues - difficulties in manufacture, scale-up problems, and cost.
Inhibition of investigator led research due to high costs
The slow pace of clinical trials
Regulation - lack of approval for non-research use.
Community - treatments not believed to be useful by other clinicians.
Reimbursement - insufficient reimbursement to cover costs of providing treatment.
Infrastructure - lack of clinical and laboratory facilities to make use of these products.
Patient attitude and preference - perceived dislike of cell therapies in patient population.
Patient characteristics - products not applicable to majority of patient population.
Lack of Knowledge - not previously aware of the availability of these treatments.
Practicality of Use - difficulties in use of products in clinical environment.
Cost effectiveness - too costly for demonstrated benefit achieved.
Trial Problems - concerns regarding conduct and design of clinical trials to date.
Efficacy - lack of cumulative clinical trial data to justify widespread adoption.
Efficacy - lack of Phase I/II trial data to justify conducting a larger trial.
Safety - lack of proven safety data for these types of treatments.
0% 10% 20% 30% 40% 50% 60% 70% 80% 90% 100%
1 = No Barrier
2
3 = Moderate Barrier
4
5 = Significant Barrier
